# Baropodometric analyses of patients before and after bariatric surgery

**DOI:** 10.6061/clinics/2015(11)05

**Published:** 2015-11

**Authors:** Ivan Leo Bacha, Fernanda Antico Benetti, Júlia Maria D'Andréa Greve

**Affiliations:** I Faculdade de Medicina da Universidade de São Paulo, Fisiopatologia Experimental, São Paulo/SP, Brazil; II University of Alberta (UofA), Alberta Internationally Educated Physiotherapists Bridging Program, Edmonton/AB, Canada; III Faculdade de Medicina da Universidade de São Paulo, Departamento de Ortopedia e Traumatologia, Laboratório para o Estudo do Movimento, São Paulo/SP, Brazil

**Keywords:** Obesity, Morbid, Biomechanics, Foot, Body weight, Bariatric Surgery, Gait

## Abstract

**OBJECTIVE:**

The aim of this study was to evaluate the vertical component of the ground reaction force, plantar pressure, contact area of the feet and double-support time using static and dynamic (gait) baropodometry before and after bariatric surgery.

**METHODS:**

Sixteen individuals with a body mass index of between 35 and 55 were evaluated before and after bariatric surgery. Thirteen patients (81.3%) were female and three (18.8%) male and their average age was 46±10 (21-60) years. An FSCAN system (version 3848) was used for baropodometric analyses (1 km/h and 3 km/h). The peak plantar pressure and ground reaction force were measured for the rear foot and forefoot. The double-support time and foot contact area were measured during gait.

**RESULTS:**

There were reductions in the ground reaction force in the forefoot and rear foot and in the foot contact area in all evaluations and of the double-support time at 3 km/h, as well as a significant reduction in the body mass index at six months post-surgery. The peak pressure did not vary at 1 km/h and at 3 km/h, reductions in peak pressure were observed in the left and right rear feet and left forefoot.

**CONCLUSIONS:**

Weight loss after bariatric surgery resulted in decreases in the ground reaction force and contact area of the foot. Plantar pressure was decreased at 3 km/h, especially in the forefoot. There was an increase in rhythm because of a reduction in the double-support time at 3 km/h.

## INTRODUCTION

Obesity is a chronic disease characterized by excessive accumulation of fat in the body 1,2. Overweight in combination with poor body alignment results in changes in load distribution and pressure on articular surfaces, causing muscle overload and contributing to joint degeneration 3-5. The main area of absorption and power dissipation in the foot is the longitudinal arch 6, which can become overwhelmed by increasing body weight.

According to Frey and Zamora (2007), most obese subjects complain of pain in the feet and ankles that is usually related to mechanical stress caused by excess weight 7. Weight gain in both men and women increases plantar pressure, which is associated with foot pain 8.

Baropodometry involves quantitative evaluation of the functioning of the foot by measuring plantar pressure in the gait and orthostatic states. Flexible insoles with sensors that respond to the mechanical deformation caused by the vertical component of the ground reaction force 9 are used in this evaluation. Baropodometry allows for the collection of data in real time, including data on plantar pressure and functioning of the foot during gait 10,11.

The overload on the musculoskeletal system of obese individuals predisposes them to abnormal gait patterns, including a loss of mobility, low cadence and imbalance, and these patterns are directly linked to diseases of the foot, such as osteoarthritis, tendonitis, fasciitis, and even diabetic foot complications 6,. Lai et al. evaluated the three-dimensional gaits of obese adults and normal individuals and determined that the obese group had a slower gait, shorter stride length, increased stance phase and double support 17.

The surgical treatment of obesity is indicated in patients with a body mass index (BMI) of greater than 40 kg/m^2^ or of 35 kg/m^2^ in the presence of comorbidities. The goals of surgical treatment are not only weight reduction but also improvement of comorbidities and quality of life 18. Obese patients undergoing stomach reduction surgery experience a sharp decrease in body weight over a short period of time that can modify proprioception and lead to changes in posture, alignment, balance and muscle flexibility 18. Weight loss also contributes to changes in posture, body image and gait 19-20.

Although the reduction of obesity is a factor for improved health, rapid modification of the human body may require a period of adaptation to the new conditions and evaluation of gait using baropodometry can be useful to determine the impact of rapid weight loss on the feet of obese patients 21. The aim of this study was to evaluate the vertical component of the ground reaction force, plantar pressure, contact area of the feet and double-support time using static and dynamic (gait) baropodometry before and after bariatric surgery.

## METHODS

Sixteen patients of both genders were assessed at the Bariatric and Metabolic Surgical Unit of the Hospital das Clínicas at the University of São Paulo School of Medicine. The evaluations were performed immediately before and at six months after surgery. The inclusion criteria were as follows: provision of written informed consent; an age of between 20 and 60 years; a BMI of between 35 and 55 kg/m^2^; a cognitive level high enough to understand the procedures and follow the guidelines provided; and identification as an independent household walker. Patients who were unable to perform the tests were excluded.

All participants signed the consent form and the study was approved by the CAPPesq of the HCFMUSP (no. 0860/09).

The sample consisted of 16 individuals who underwent bariatric surgery, including 13 (81.3%) females and three (18.8%) males. The average age was 46±10 (21-60) years.

Initially, a clinical review was conducted that included anthropometric measurements of body mass (kg) and height (m) in patients wearing only swimsuits, the use of the Feiss line to assess the extent of the medial longitudinal plantar arch and assessment of the BMI.

**Figure 1:**
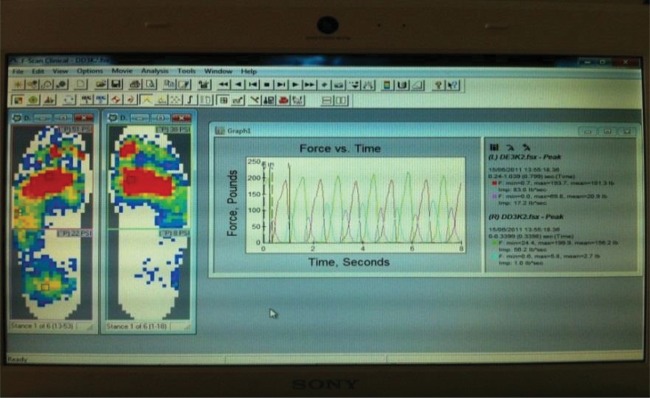
FSCAN system version 3848, which measures the peak values for pressure and the ground reaction force for the rear foot and forefoot, the double-support time, and the foot contact area.

**Figure 2:**
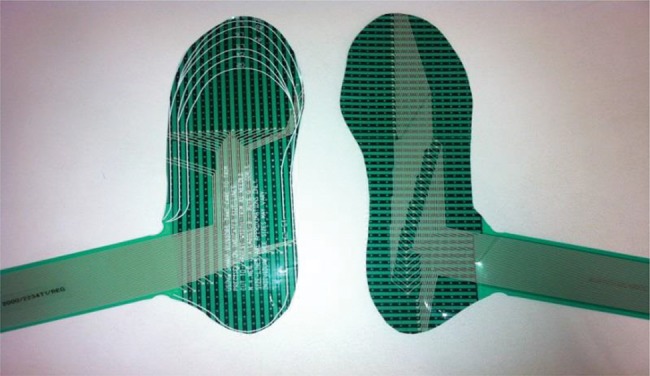
Insoles. Each insole was 0.18 mm thick and had 960 sensors that were sensitive to mechanical strain and were evenly distributed every five millimetres as a screen over the entire surface of the foot.

After the clinical evaluation, all volunteers were subjected to static and dynamic baropodometric assessments using an FSCAN system (Figure 1) version 3848, which measures the peak values of pressure and the ground reaction force in the rear foot and forefoot, the double-support time and the foot contact area. Flexible insoles were cut according to the sizes of each volunteer's feet (Figure 2) and were placed inside of the shoes. Each insole was 0.18 mm thick and had 960 sensors that were sensitive to mechanical strain. The sensors were evenly distributed as a screen over the entire surface of the foot.

Static and dynamic acquisitions were performed (1 km/h and 3 km/h) and each dynamic acquisition captured six to seven steps. We selected the four core steps of each acquisition for analysis.

### Statistical analysis

The subjects were characterized with regard to age and gender. Foot types, as determined according to the Feiss line, were analysed using McNemar's test.

The distributions of the quantitative variables in both the initial and final evaluations (six months after surgery) were normal, permitting application of the paired t-test. The Shapiro-Wilk distribution of normality test was also conducted. The peak of the static ground reaction force in the right rear foot, peak ground reaction force (3 km/h) in the right forefoot and pressure peak were not normally distributed and non-parametric Wilcoxon post-test was used for paired samples.

## RESULTS

The BMIs before and after surgery are shown in Table 1. There was a significant difference in the BMI between the two assessments (*p*<0.001).

The results for the vertical component of the ground reaction force before and after bariatric surgery are depicted in Table 2, which shows the differences between the first and second evaluations, with a peak reduction in the ground reaction force at six months after bariatric surgery in all individuals.

The results for the plantar contact areas before and after bariatric surgery are depicted in Table 3, which shows that there was a reduction in the plantar contact area at six months after bariatric surgery in all subjects.

The descriptive statistical values for peak plantar pressure are shown in Table 4. There were reductions in plantar pressure at six months after surgery in the right forefoot (*p*=0.016), right rear foot (*p*=0.010), left forefoot (*p*=0.034), and left rear foot (*p*=0.026). There was no plantar pressure reduction at 1 km/h at six months after surgery. The results were not homogeneous at 3 km/h because there was no plantar pressure reduction in the right forefoot (*p*=0.133) but there were reductions at six months after surgery in the right rear foot (*p*=0.047), left forefoot *(p*=0.044) and left rear foot (*p*=0.036).

The descriptive statistical values for the double-support time parameter in the initial and final evaluations are shown in Table 5. There was no significant difference in the evaluations performed at 1 km/h (*p*=0.434). At 3 km/h, the double-support time for the final evaluation was less than that at the initial evaluation (*p*=0.003).

## DISCUSSION

The results showed that the loss of body weight at six months after bariatric surgery caused reductions in the value of the vertical component of the ground reaction force and the area of plantar support in all assessments of the forefoot and rear foot but that it resulted in a less marked reduction in plantar pressure, which is more dependent on the area and running speed. These results show that a significant reduction in the load applied to the feet occurs in patients with morbid obesity when there is a loss of body weight and a decrease in the BMI.

In the static baropodometry, the plantar pressure peak, ground reaction force and contact area of the foot are greater than those of non-obese individuals, according to Fabris et al. (2006) and Birtane and Tuna (2004). These results are in partial agreement with those of the current study, in which all of the patients showed decreased plantar pressure in the forefoot and rear foot in the static evaluation following a loss of body mass as a result of bariatric surgery. Evaluation of foot morphology, which was accomplished by tracing the Feiss line, showed no difference between the initial assessment and that performed six months later, indicating that the loss of body mass did not result in modification of the foot type. Thus, the reductions in plantar pressure were related to body mass loss and not to foot shape.

The dynamic evaluation, which was performed at two speeds, was pre-determined to be suitable for use under the initial patient conditions, and it revealed not only reductions in the ground reaction force and contact area but also in the distinct pattern of plantar pressure.

The reaction forces in the gait at 1 km/h were significantly decreased in the forefoot and rear foot of both feet, and no significant reduction in plantar pressure was observed, indicating that greater variability in pressure and its dependence on other factors are more important in the dynamic evaluation. Plantar pressure is defined as the ground reaction force divided by the area of application of this force, i.e., the plantar contact area. This pressure can be considered an indirect data point because it is calculated from the ground reaction force and contact area; however, the decrease in both values could result in no reduction in plantar pressure due to a smaller force distribution in a smaller contact area.

The ground reaction force was significantly decreased at 3 km/h in both feet and in both regions studied and plantar pressure was reduced at all measured sites, with the exception of the right forefoot, whose values did not differ from those observed at the lower speed. It is possible that the largest effect of body mass reduction on plantar pressure occurred at the higher speed, even when both of these parameters were decreased. The small sample size may have contributed to the differing results obtained at the two speeds, but other factors, such as deformities, rigidity and gait pattern changes, can interfere with plantar pressure because the condition of the plantar surface has a greater effect on plantar pressure than the ground reaction force.

In addition to body mass, mobility is an important factor for proper plantar pressure distribution. Rigid feet or those with some type of deformity are more susceptible to hyper-pressure areas, changes in gait and pain. Reducing foot mobility interferes with the vertical component of the ground reaction force in normal feet and can cause pain and structural changes over time 22.

In patients with morbid obesity, increasing the support area is one of the few strategies that can help to reduce overload on the musculoskeletal structures by promoting plantar pressure reduction because the ground reaction force is affected by an individual's body mass. Decreasing gait speed, which causes an increase in the double-support time is another strategy for plantar pressure reduction, but patients with a very high BMI certainly exceed the compensatory capacity of the body for maintaining functionality.

In all measurements, the decrease in the ground reaction force was greater than that in plantar pressure, indicating that plantar pressure, calculated by dividing the vertical component of the ground reaction force by the contact area, can be more variable and susceptible to other factors, mainly to postural corrections from oscillations that are required to maintain balance in the orthostatic and gait states. This pressure is more variable, while the force is directly affected by an individual's body mass.

The limitations of this study are directly related to its small sample size, which created difficulties in achieving a homogeneous initial sample, subsequent sampling loss due to postoperative complications and variations in the body mass loss of each individual over time. However, the study still revealed some important results pertaining to the feet of patients with morbid obesity.

This study shows that the loss of body mass helps to improve the functioning of the feet in terms of support and locomotion but indirectly reveals that other factors, such as the muscular condition, morphology and mobility of the feet, need to be considered in this assessment, particularly in relation to future rehabilitation interventions. Increasing the capacity of locomotion in patients with morbid obesity is an important factor for improving the quality of life and success of bariatric surgery.

The following modifications were observed in the static and dynamic baropodometric parameters in the patients with morbid obesity at six months after bariatric surgery: reductions in the ground reaction force and the plantar support area for all subjects, reductions in plantar pressure in the static evaluation and at 3 km/h, and a reduction in the double-support time at 3 km/h.

## Figures and Tables

**Table 1:** Descriptive statistics for body mass index (kg/m^2^) at the time of initial surgery (early time) and at 6 months after surgery.

Assessment	N	Average	SD	Minimum	Maximum	*p*
Initial (before surgery)	16	*44.6	4.5	35	52.5	<0.001
Final (6 months after)						16	*32.6	2.7	29.2	37.6	<0.001

SD = standard deviation; Paired t-test

**Table 2:** Descriptive statistics for the vertical component of the static and dynamic (1 and 3 km/h) ground reaction force (lb) measured at initial (initial) and final (six months after bariatric surgery) evaluations of the right and left feet, for the forefeet and rear feet of 16 volunteers.

	Side	Region	Evaluation	Average	SD	Median	Minimum	Maximum	*p*
Static	Right	Forefoot	Initial	*44.7	25	38.4	12.1	http://dx.doi.org/101.2	0.019
			6 months	*33.3	21.4	36	0.8	67.2	
		Rear foot	Initial	87.5	17	*88.8	61.5	130	<0.001
			6 months	52.3	17.9	*56.6	12.8	75.5	
	Left	Forefoot	Initial	*44.3	26.5	45.9	7.2	http://dx.doi.org/105.6	0.046
			6 months	*34.2	15.5	35.8	12.5	64.6	
		Rear foot	Initial	*72.2	19	74.8	31.4	http://dx.doi.org/100.4	0.001
			6 months	*49.3	18.3	53.7	18.9	75.1	
1 km/h	Right	Forefoot	Initial	*139.2	63.2		51.7	281.3	0.001
			6 months	*103	39.3		41.4	172.9	
		Rear foot	Initial	*146.8	38.8		91	221.4	0.003
			6 months	*118.9	28.3		61.9	165.7	
	Left	Forefoot	Initial	*143.4	55.2		56.6	239	0.005
			6 months	*118.2	52.2		48	262	
		Rear foot	Initial	*140.0	40		48.3	194.1	0.026
			6 months	*115.7	29.6		64.1	186.8	
3 km/h	Right	Forefoot	Initial	194.7	47.9	*188.4	94	270.4	<0.001
			6 months	156.2	35.9	*144.8	93.8	220.8	
		Rear foot	Initial	*130.6	33.2	130.6	76.2	193.5	0.002
			6 months	*105.1	25.4	http://dx.doi.org/101.9	73.9	153.3	
	Left	Forefoot	Initial	*209.9	54.7	210.1	123.4	319.3	<0.001
			6 months	*156.2	39	145	http://dx.doi.org/105.1	269	
		Rear foot	Initial	*129.9	26.6	127.1	81	173.7	0.001
			6 months	*100.3	30.6	95.8	37.7	162.7	

SD= Standard Deviation; Paired t-test (Average) and Wilcoxon's Test (Median)

**Table 3:** Descriptive statistics for the plantar contact area (cm^2^) under static and dynamic conditions (1 and 3 km/h) at the initial (initial) and final (six months after bariatric surgery) evaluations of the right and left feet, for the forefeet and rear feet of 16 volunteers.

	Side	Evaluation	Average	SD	Minimum	Maximum	*p*
Static	Right	Initial	*91.5875	18.6413	63.74	128	0.002
		6 months	*68.9013	26.606	31.48	115.35	
	Left	Initial	87.6125	22.8014	52.13	125.42	0.056
		6 months	71.4519	18.3563	31.74	http://dx.doi.org/104.77	
1 km/h	Right	Initial	*108.014	17.9792	67.61	146.58	0.01
		6 months	*92.4675	17.0209	63.48	128.77	
	Left	Initial	*111.273	17.6693	82.84	142.45	0.004
		6 months	*94.8063	19.8817	60.13	138.32	
3 km/h	Right	Initial	*102.904	18.5105	59.87	137.55	0.009
		6 months	*88.935	16.1116	59.87	123.61	
	Left	Initial	*104.999	15.2759	75.61	131.35	0.001
		6 months	*84.9175	18.2079	59.35	128	

SD= Standard Deviation; Paired t-test

**Table 4:** Descriptive statistics for the peak pressure under static and dynamic conditions (1 and 3 km/h) measured at the initial (initial) and final (six months after bariatric surgery) evaluations for the right and left feet, for the forefeet and rear feet of 16 volunteers.

	Side	Region	Evaluation	Median	Minimum	Maximum	*p*
Static	Right	Forefoot	Initial	*15	5	55	0.016
			6 months	*11	5	20	
		Rear foot	Initial	*23.5	19	56	0.01
			6 months	*16	11	40	
	Left	Forefoot	Initial	*14	5	21	0.034
			6 months	*11	7	36	
		Rear foot	Initial	*23	15	35	0.026
			6 months	*17	8	36	
1 km/h	Right	Forefoot	Initial	38	5	55	0.313
			6 months	36	5	20	
		Rear foot	Initial	43.5	19	56	0.147
			6 months	43	11	40	
	Left	Forefoot	Initial	42.5	5	21	0.352
			6 months	35.5	7	36	
		Rear foot	Initial	41.5	15	35	0.604
			6 months	37	8	36	
3 km/h	Right	Forefoot	Initial	67.5	48	201	0.133
			6 months	59	41	121	
		Rear foot	Initial	*45.5	23	http://dx.doi.org/107	0.047
			6 months	*34.5	20	74	
	Left	Forefoot	Initial	*79.5	56	145	0.044
			6 months	*64	48	191	
		Rear foot	Initial	*44.5	22	64	0.036
			6 months	*34	14	65	

Wilcoxon's Test

**Table 5:** Descriptive statistics for the double-support time (s) at the initial and final (six months after surgery) evaluations at 1 km/h and 3 km/h.

Speed	Evaluation	Average	SD	Minimum	Maximum	*p*
1 km/h	Initial	0.4	0.1	0.22	0.54	0.434
	6 months	0.38	0.1	0.18	0.58	
3 km/h	Initial	*0.13	0.02	0.1	0.16	0.003
	6 months	*0.11	0.02	0.08	0.16	

SD= Standard Deviation; Paired t-test
